# Association of Insurance Status With Provision of Recommended Services During Comprehensive Postpartum Visits

**DOI:** 10.1001/jamanetworkopen.2020.25095

**Published:** 2020-11-10

**Authors:** Kimberley Geissler, Brittany L. Ranchoff, Michael I. Cooper, Laura B. Attanasio

**Affiliations:** 1 Department of Health Promotion and Policy, University of Massachusetts Amherst School of Public Health and Health Sciences, Amherst; 2 University of Massachusetts Amherst School of Public Health and Health Sciences, Amherst

## Abstract

**Importance:**

Improving care during the postpartum period is a clinical and policy priority. During the comprehensive postpartum visit, guidelines recommend delivery of a large number of assessment, screening, and counseling services. However, little is known about services provided during these visits.

**Objective:**

To examine rates of recommended services during the comprehensive postpartum visits and differences by insurance type.

**Design, Setting, and Participants:**

This cross-sectional study included 20 071 093 weighted office-based postpartum visits (645 observations) with obstetrical-gynecological or family medicine physicians from annual National Ambulatory Medical Care Surveys from December 28, 2008, to December 31, 2016, and estimated multivariate regression models to calculate the frequency of recommended services by insurance type, controlling for visit, patient, and physician characteristics. Data analysis was conducted from November 1, 2019, to September 1, 2020.

**Exposures:**

Visit paid by Medicaid vs other payment types.

**Main Outcomes and Measures:**

Visit length and binary indicators of blood pressure measurement, depression screening, contraceptive counseling or provision, pelvic examinations, Papanicolaou tests, breast examinations, medication ordered or provided, referral to other physician, and counseling for weight reduction, exercise, stress management, diet and/or nutrition, and tobacco use.

**Results:**

A total of 20 071 093 weighted comprehensive postpartum visits to office-based family medicine or obstetrical-gynecological physicians were included (mean patient age, 29.7 [95% CI, 29.1-30.3] years). Of these visits, 34.3% (95% CI, 27.6%-41.1%) were covered by Medicaid. Mean visit length was 17.4 (95% CI, 16.4-18.5) minutes. The most common procedures were blood pressure measurement (91.1% [95% CI, 88.0%-94.2%]), pelvic examinations (47.3% [95% CI, 40.8%-53.7%]), and contraception counseling or provision (43.8% [95% CI, 38.2%-49.3%]). Screening for depression (8.7% [95% CI, 4.1%-12.2%]) was less common. When controlling for visit, patient, and physician characteristics, the only significant difference in visit length or provision of recommended services based on insurance type was a difference in provision of breast examinations (14.7% [95% CI, 8.0%-21.5%] for Medicaid vs 25.6% [95% CI, 19.4%-31.8%] for non-Medicaid; *P* = .02).

**Conclusions and Relevance:**

These findings suggest that receipt of recommended services during comprehensive postpartum visits is less than 50% for most services and is similar across insurance types. These findings underscore the importance of efforts to reconceptualize postpartum care to ensure women have access to a range of supports to manage their health during this sensitive period.

## Key Points

**Question:**

How frequently are specific recommended services provided during comprehensive postpartum visits, and are there differences by insurance type?

**Findings:**

This cross-sectional study of a weighted 20 071 093 postpartum office visits found most women, including those with and without Medicaid insurance, did not receive the full set of recommended services and counseling during their comprehensive postpartum visit.

**Meaning:**

These findings suggest that substantial room exists to improve the delivery of postpartum care.

## Teaser

This cross-sectional study examines rates of recommended services during comprehensive postpartum visits and differences between Medicaid and non-Medicaid insurance type among US women.

## Introduction

In the United States, approximately 4 million births occur annually.^1,2,3^ The postpartum period is a critical time for maternal and infant well-being as a woman adjusts to physical, social, and psychological changes. The postpartum period presents a unique opportunity to promote health while preventing^4^ and managing^5,6,7^ chronic diseases for women. The typical postpartum care sequence in the United States traditionally consisted of a single visit at 4 to 6 weeks after delivery.^8^ Recent American College of Obstetricians and Gynecologists (ACOG) guidance proposes reconceptualizing postpartum care as a continuum rather than a single visit; however, a comprehensive postpartum visit by 12 weeks after delivery is still recommended in addition to other clinician contact during the postpartum period.^6^

Significant research has focused on women’s comprehensive postpartum visit attendance; estimates of nonattendance vary widely, from 11% to 50%, with higher nonattendance rates among higher-risk populations.^6,7,8,9,10,11,12,13,14,15,16^ Women with limited resources,^11,12,13,14,17^ those who do not attend prenatal care visits,^14,15,17^ and those who perceived discrimination during the childbirth hospitalization^18^ are less likely to attend a postpartum visit. Increasing postpartum visit attendance has been a focus of quality improvement as a Healthy People 2020 goal and a Healthcare Effectiveness Data and Information Set metric.^8,19,20^

Despite attention to use of postpartum visits, little is known about what services are actually provided during the comprehensive postpartum visit. Previous ACOG guidance noted clinicians should conduct a “full assessment of physical, social, and psychological well-being” as well as provide counseling and the full range of US Food and Drug Administration–approved contraceptive methods during the comprehensive visit.^8^^(p187)^ The ACOG’s 2018 guidance more specifically details the assessment, screening, and counseling services that should be provided during the comprehensive postpartum visit.^6^ If these services are not provided, even women who attend the postpartum visit may be left with unmet clinical needs and concerns and may not effectively transition to ongoing care after this period. In a recent survey, postpartum clinicians self-reported nearly always addressing pregnancy and birth complications, screening for depression, and contraceptive counseling.^21^ Survey findings also indicated a disconnect between clinicians’ perceptions of service importance and how frequently those services were delivered. For example, pelvic examinations were not rated as very important but were usually performed during postpartum visits.^21^

Although this recent survey provides information about clinicians’ perceptions of care delivery, little is known about whether recommended services are provided during the comprehensive postpartum visit and whether these services vary by patient characteristics, such as insurance type. Owing to higher income eligibility thresholds for Medicaid coverage during pregnancy, 43% to 45% of US births were covered by Medicaid from 2008 to 2016.^3,22^ However, Medicaid eligibility for pregnancy only extends through 60 days after delivery nationally; even in states that expanded Medicaid under the Patient Protection and Affordable Care Act, 30% of women experience insurance changes or disruptions in the perinatal period.^23^ Receiving the full set of recommended services in the postpartum period may be even more important for women who lose Medicaid eligibility. However, prior studies^24,25,26^ have found that women with Medicaid receive fewer preventive services during clinical encounters compared with privately insured women.

A better understanding of which services are commonly delivered during comprehensive postpartum care visits will provide guidance in ongoing efforts to improve the quality of maternity care services, and postpartum care specifically, for US women. The goal of this study was to use national visit-level data to document the frequency of recommended services during postpartum visits and to examine potential differences in the provision of services by patient insurance status. We hypothesized that recommended services would be less frequently provided during postpartum visits for women with Medicaid insurance compared with women with other coverage types.

## Methods

### Overview

Using the National Ambulatory Medical Care Survey (NAMCS) and a cross-sectional design, we estimated the frequency of ACOG-recommended procedures and screening for women during a comprehensive postpartum visit. We analyzed differences in care received by insurance type, controlling for factors associated with use of preventive screening services. This study was reviewed by the University of Massachusetts institutional review board and determined to be not human participants research, which did not require informed consent given the use of deidentified data. This study followed the Strengthening the Reporting of Observational Studies in Epidemiology (STROBE) reporting guideline.^27^

### Data and Analytic Sample

We use data on ambulatory care visits to office-based physicians from annual NAMCS surveys for December 28, 2008, to December 31, 2016, conducted by the National Center for Health Statistics. The NAMCS surveys physicians annually and includes detailed information abstracted from clinical records for 50 patient visits in a 1-week period. The National Center for Health Statistics constructs visit weights to correct for physician selection and nonresponse^28^; these weights make the sample nationally representative of office-based physician visits.

The analytic sample includes all comprehensive postpartum visits identified using code V24.2 from the *International Classification of Diseases, Ninth Revision* and code V39.2 from the *International Statistical Classification of Diseases and Related Health Problems, Tenth Revision* as well as primary reason for visit codes classified by the National Center for Health Statistics (3215.0).^28,29^ We limited the sample to visits for women aged 18 to 44 years with family medicine or obstetrical/gynecological (ob/gyn) physicians. Based on NAMCS inclusion criteria, the sample included visits to office-based physicians, including physicians employed by hospital-owned practices in 2014 to 2016. The sample excluded physicians practicing in community health centers and hospital outpatient facilities and federally employed physicians^28^; it did not include certified nurse-midwives practicing independently. We also excluded observations missing data on control variables.

### Measures

The primary outcome measures are services recommended as part of a comprehensive postpartum visit as well as other services that may occur. Table 1 shows the correspondence between available measures in the NAMCS and ACOG recommendations. Recommended services include blood pressure screening; depression screening; pelvic examination; Papanicolaou test; breast examination; blood glucose level examination; contraceptive counseling or provision; counseling for weight reduction, exercise, stress management, diet and nutrition, and/or tobacco use; medication ordered or provided; and referral to another physician. These variables are recorded in the NAMCS using clinical abstraction of the medical record and were consistently available during the full period of the data. Contraceptive counseling or provision includes visits with family planning counseling, contraceptive medication provided, or reason for visit, diagnosis, or procedure codes related to contraceptives (eTable 1 in the Supplement). We report measurement of blood glucose levels and counseling about tobacco use in the descriptive statistics only. A fasting blood glucose level measurement or oral glucose tolerance test is only recommended for women who experience gestational diabetes, and tobacco use counseling is limited to women who use tobacco. The primary independent variable is whether the visit was paid by Medicaid vs other payment types, including private insurance, self-pay, and other.

**Table 1.** Recommended Services in a Comprehensive Postpartum Visit^a^

Component	Recommended services and anticipatory guidance	Corresponding measure(s) in NAMCS
Mood and emotional well-being	Screen for postpartum depression Screen for/counsel on tobacco use Screen for substance use disorder Guidance on mentoring and support Counsel on preexisting mental health disorders	Depression screening Tobacco use counseling
Infant care and feeding	Assess comfort and confidence with caring for newborn Assess comfort and confidence with breastfeeding Assess material needs	NA
Sexuality, contraception, and birth spacing	Assess reproductive life plan Select contraceptive method Guidance on sexuality, dyspareunia, and resumption of intercourse Explain risks/benefits of pregnancy within 6 and 18 mo Counsel on prevention of recurrent pregnancy complications	Family planning counseling Pelvic examination Diagnosis and procedure codes for contraceptives
Sleep and fatigue	Review options to cope with fatigue and sleep disruption Advise on family and friends assistance with care responsibilities	Stress management counseling
Physical recovery from birth	Counseling on physical activity and weight management Assess presence of perineal or cesarean incision pain and appropriate guidance Assess presence of urinary and fecal continence, referral if indicated	Diet/nutrition counseling Exercise counseling Weight reduction counseling
Chronic disease management	Glucose screening if gestational diabetes Refer for follow-up care with PCP or subspecialist clinicians as indicated Review medication regimen Discuss pregnancy complications and implications for future childbearing and maternal health	Glucose blood level test
Health maintenance	Perform well-woman screening, including Papanicolaou test and pelvic examination as indicated Review vaccinations	Blood pressure screening Pelvic examination Pap test Breast examination
		Services in multiple categories: Medication ordered or provided Referral to another physician

Abbreviations: NA, not applicable; PCP, primary care physician.

^a^ From American College of Obstetricians and Gynecologists Committee Opinion No. 736.^6^

### Statistical Analysis

Data were analyzed from November 1, 2019, to September 1, 2020. To determine whether receipt of recommended services differs between those with Medicaid vs other payment types, we estimated linear probability models for binary and continuous outcomes. We first estimated unadjusted models, including the independent variable of Medicaid coverage. We then estimated linear probability models controlling for physician, patient, and visit characteristics known to be associated with use of preventive services.^24,25,26,30,31^ Visit characteristics include Medicaid payment and a linear trend for year of visit. Patient characteristics include age, race/ethnicity, and indicators of 5 comorbidities (asthma, diabetes, depression, hypertension, and obesity). Race/ethnicity was physician reported, with imputation for missing values by the National Center for Health Statistics. Physician characteristics include specialty (ob/gyn vs family medicine), office location in a metropolitan statistical area, physician as full or part owner of a practice, private solo or group practice, and solo practice. We report both unadjusted and adjusted estimated probabilities of the outcomes.

We conducted a sensitivity analysis in which we compared Medicaid vs private insurance, which constituted most non-Medicaid visits. We also examined changes over time in the receipt of services during the comprehensive postpartum visit, controlling for whether the visit was paid for by Medicaid, and report estimated probabilities of the outcomes.

Standard errors corrected for the complex survey design. Two-sided α = .05 was considered statistically significant. All analyses were conducted in Stata MP, version 16.1 (StataCorp LLC).

## Results

**Figure 1.**
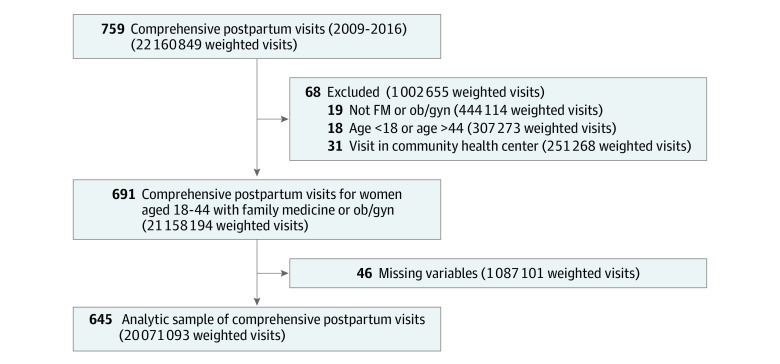
Analytic Sample Selection Ob/gyn indicates obstetrics/gynecology.

The analytic sample included 20 071 093 weighted visits (645 unweighted observations) with an office-based family medicine or ob/gyn physician, with a mean patient age of 29.7 (95% CI, 29.1-30.0) years (Figure 1, Table 2, and Table 3). Of these visits, 34.3% (95% CI, 27.6%-41.1%) were covered by Medicaid; 90.8% (95% CI, 85.0%-96.7%) were with ob/gyn physicians; and 9.2% (95% CI, 3.3%-15.0%) were with family medicine physicians. The mean length of visit was 17.4 (95% CI, 16.4-18.5) minutes. The most common procedures during these visits were blood pressure measurement (91.1% [95% CI, 88.0%-94.2%]), pelvic examinations (47.3% [95% CI, 40.8%-53.7%]), breast examinations (21.9% [95% CI, 16.8%-26.9%]), and Papanicolaou tests (15.9% [95% CI, 11.6%-20.1%]). Screening for depression (8.7% [95% CI, 4.1%-12.2%]) was less common. Among the overall visits, 43.8% (95% CI, 38.2%-49.3%) had contraceptive counseling or provision; 2.8% (95% CI, 0.9%-4.7%), counseling about weight reduction; 9.0% (95% CI, 5.4%-12.6%), counseling about exercise; and 10.1% (95% CI, 6.2%-14.0%), counseling about diet and/or nutrition. Medication was ordered or provided at 58.0% (95% CI, 50.9%-65.0%) of visits. Only 2.3% (95% CI, 1.0%-3.5%) of visits resulted in a referral to another physician.

**Table 2.** Descriptive Statistics of Services Provided at Comprehensive Postpartum Visits

Service	Patient group, % (95% CI)^a^		
	Overall (N = 20 071 093)	Medicaid payment (n = 6 892 503)	Non-Medicaid payment (n = 13 178 590)
Time spent with clinician, mean (95% CI), min	17.4 (16.4-18.5)	18.0 (16.2-19.9)	17.1 (16.0-18.2)
Blood pressure taken	91.1 (88.0-94.2)	94.2 (90.8-97.6)	89.4 (85.3-93.6)
Depression screening	8.7 (4.1-12.2)	8.0 (2.2-13.8)	9.0 (4.7-13.3)
Pelvic examination	47.3 (40.8-53.7)	37.3 (26.8-47.7)	52.5 (45.2-59.7)^b^
Papanicolaou test	15.9 (11.6-20.1)	13.6 (7.2-20.1)	17.0 (12.2-21.9)
Breast examination	21.9 (16.8-26.9)	13.8 (7.9-19.7)	26.1 (19.9-32.3)^b^
Contraceptive counseling or provision	43.8 (38.2-49.3)	43.6 (32.0-55.2)	43.9 (38.3-49.5)
Counseling			
Weight reduction	2.8 (0.9-4.7)	3.9 (0.1-7.6)	2.2 (0.1-4.3)
Exercise	9.0 (5.4-12.6)	10.6 (1.7-19.6)	8.2 (4.7-11.7)
Stress management	4.7 (1.3-8.0)	7.4 (-1.2-15.9)	3.3 (0.7-5.9)
Diet and/or nutrition	10.1 (6.2-14.0)	10.4 (1.6-5.6)	10.0 (5.6-14.4)
Tobacco use	2.3 (0.5-4.0)	3.0 (0.5-5.4)	1.9 (-0.6-4.4)
Glucose blood level test	0.9 (-0.2-2.1)	0.5 (-0.08-1.0)	1.1 (-0.6-2.8)
Medication ordered or provided^c^	58.0 (50.9-65.0)	59.2 (46.5-72.0)	57.3 (50.3-64.4)
Refer to other physician^d^	2.3 (1.0-3.5)	1.2 (-0.04-2.4)	2.8 (1.1-4.6)

^a^ Overall includes 645 observations; Medicaid payment, 205 observations; and non-Medicaid payment, 440 observations.

^b^ Indicates difference between visits covered by Medicaid vs other payment types is statistically significant with *P* < .05.

^c^ Includes 20 013 231 weighted visits and 642 observations.

^d^ Includes 19 397 174 weighted visits and 627 observations.

**Table 3.** Descriptive Statistics of Patients and Clinicians at Comprehensive Postpartum Visits

Characteristic	Patient group, % (95% CI)^a^		
	Overall (N = 20 071 093)	Medicaid payment (n = 6 892 503)	Non-Medicaid payment (n = 13 178 590)
**Clinician **			
Ob/gyn (vs family medicine)	90.8 (85.0 to 96.7)	84.7 (70.6 to 98.7)	94.0 (89.9 to 98.2)
Practice uses any electronic medical records	73.4 (67.1 to 79.8)	76.1 (66.0 to 86.2)	72.0 (64.8 to 79.3)
In metropolitan statistical area	88.6 (82.4 to 94.9)	85.8 (71.8 to 99.8)	90.1 (85.2 to 95.0)
Physician is full or part owner of practice	66.2 (58.5 to 73.9)	70.0 (55.9 to 84.0)	64.2 (56.2 to 72.3)
Office setting is a private solo or group practice	88.3 (79.3 to 97.3)	88.7 (76.0 to 101.4)	88.1 (80.1 to 96.1)
Physician is in a solo practice	29.4 (21.7 to 37.1)	43.2 (29.2 to 57.2)	22.2 (14.7 to 29.8)^b^
**Patient **			
Age, mean (95% CI), y	29.7 (29.1 to 30.3)	26.7 (25.8 to 27.6)	31.2 (30.5 to 31.9)^b^
Race/ethnicity			
Non-Hispanic White	61.3 (54.5 to 68.1)	44.7 (32.4 to 57.0)	70.0 (63.4 to 76.7)^b^
Non-Hispanic Black	12.4 (8.6 to 12.3)	21.8 (13.2 to 30.3)	7.6 (4.7 to 10.4)^b^
Hispanic	19.8 (14.3 to 25.3)	31.5 (19.1 to 44.0)	13.6 (9.4 to 17.9)^b^
Non-Hispanic other	6.5 (3.0 to 9.9)	2.0 (−0.9 to 5.0)	8.8 (3.9 to 13.7)^b^
Visit payment type			
Medicaid	34.3 (27.6 to 41.1)	100	0^b^
Private insurance	61.3 (54.9 to 67.7)	0	93.3 (90.6 to 96.0)^b^
Other	4.4 (25.3 to 6.3)	0	6.7 (4.0 to 9.4)^b^
Patient comorbidities			
Asthma	4.5 (2.0 to 7.0)	5.2 (−0.2 to 10.7)	4.1 (1.3 to 7.0)
Diabetes	4.1 (1.5 to 6.6)	3.0 (−0.4 to 6.4)	4.7 (1.3 to 8.0)
Depression	7.0 (4.2 to 9.8)	9.6 (3.6 to 15.6)	5.6 (2.6 to 8.7)
Hypertension	6.9 (4.0 to 9.8)	9.3 (2.9 to 15.7)	5.7 (2.5 to 8.8)
Obesity	5.3 (2.8 to 7.9)	6.7 (1.9 to 11.6)	4.6 (1.9 to 7.3)

Abbreviation: Ob/gyn, obstetrics/gynecology.

^a^ Overall includes 645 observations; Medicaid payment, 205 observations; and non-Medicaid payment, 440 observations.

^b^ Indicates difference between visits covered by Medicaid vs other payment types is statistically significant with *P* < .05.

**Figure 2.**
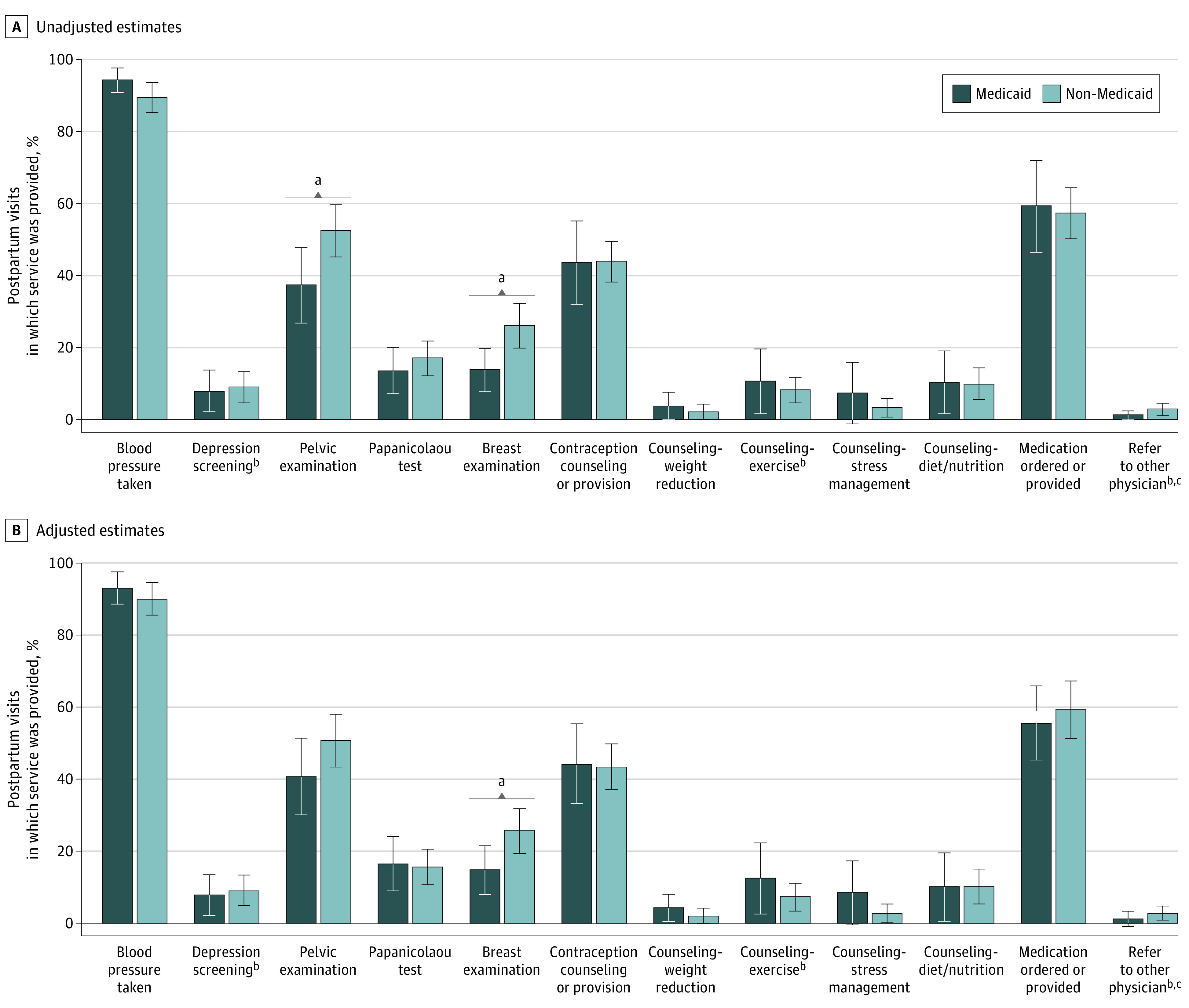
Services Provided During Comprehensive Postpartum Visits (2009-2016) Vertical bars represent 95% CIs. Standard errors correct for the complex survey design. Sample size is 20 071 093 weighted visits (645 unweighted observations). National Ambulatory Medical Care Survey notes that National Center for Health Statistics does not consider estimates relying on fewer than 30 observations and/or with standard errors greater than 30% of estimates to be reliable. Referral to other physician has 19 397 174 weighted visits (627 unweighted observations). Medication ordered or provided has 20 013 231 weighted visits (642 unweighted observations). A, Regression estimates of probability of service occurring during visit are reported. B, Regression-adjusted estimates of probabilities are reported; controls are included for whether the visit was paid by Medicaid, year of visit, patient age, patient race/ethnicity, patient comorbidities (asthma, diabetes, depression, hypertension, and obesity), physician specialty (obstetrics/gynecology vs family medicine), office location in a metropolitan statistical area, physician as full or part owner of practice, private solo or group practice, and solo practice. ^a^Difference between Medicaid and non-Medicaid estimates is statistically significant with *P* < .05. ^b^Estimates for Medicaid unadjusted estimates have standard errors (shown as part of the 95% CI) that exceed the threshold of greater than 30% of estimates (reported for completeness). ^c^Estimates for non-Medicaid unadjusted estimates have standard errors (shown as part of the 95% CI) that exceed the threshold of greater than 30% of estimates (reported for completeness).

Few significant differences were found in the services provided at comprehensive postpartum visits between those with and without Medicaid. In unadjusted analyses, pelvic examinations (52.5% [95% CI, 45.2%-59.7%] for non-Medicaid vs 37.3% [95% CI, 26.8%-47.7%] for Medicaid; *P* = .01) and breast examinations (26.1% [95% CI, 19.9%-32.3%] for non-Medicaid vs 13.8% [95% CI, 7.9%-19.7%] for Medicaid; *P* = .003) were statistically significantly more common among those with non-Medicaid payment types than those with Medicaid (Figure 2A).

After controlling for visit, patient, and physician characteristics, there were few statistically significant differences in services provided based on insurance type (Figure 2B; eTable 2 in the Supplement). Breast examinations were again less common among visits paid by Medicaid (14.7% [95% CI, 8.0%-21.5%] for Medicaid vs 25.6% [95% CI, 19.4%-31.8%] for non-Medicaid; *P* = .02), but no other differences were statistically significant. Time spent with a physician during the visit was similar between insurance types (mean, 17.7 [95% CI, 15.9-19.6] minutes for Medicaid vs 17.3 [95% CI, 16.1-18.5] minutes for non-Medicaid).

In the sensitivity analysis in which we compared visits paid by Medicaid and private insurance, results were similar (eFigure 1 in the Supplement). In the second sensitivity analysis analyzing changes over time, changes were not consistent across services. Breast examinations (30.7% [95% CI, 22.2% to 39.2%] for 2009 to 2010 vs 16.6% [95% CI, 6.0% to 27.3%] for 2015 to 2016; *P* = .04), Papanicolaou tests (28.9% [95% CI, 19.6% to 38.2%] for 2009 to 2010 vs 6.0% [95% CI, −1.5% to 13.5%] for 2015 to 2016; *P* < .001), and contraception counseling or provision (50.8% [95% CI, 39.6% to 61.9%] for 2009 to 2010 vs 34.3% [95% CI, 24.1% to 44.5%] for 2015 to 2016; *P* = .03) were significantly less common in 2015 to 2016 than in 2009 to 2010, whereas blood pressure measurement (85.8% [95% CI, 79.0% to 92.7%] for 2009 to 2010 vs 95.3% [95% CI, 90.3% to 100.9%] for 2015 to 2016; *P* = .02) was more common (eFigure 2 in the Supplement).

## Discussion

Using national data abstracted from clinical records for 2009 through 2016, we found that receipt of recommended services during the comprehensive postpartum visit was less than 50% for most procedures, with 1 in 11 visits including depression screening. The receipt of these services was similar across insurance types, rather than being lower among low-income women covered by Medicaid, as previous studies of preventive services have found.^24,25,32^ We examined services that should be universally or nearly universally provided, including depression screening, contraceptive counseling, and blood pressure monitoring, as well as services only indicated for some women, such as tobacco use counseling or measurement of blood glucose levels. The lack of recommended service provision may also reflect the long list of recommended services^6^ combined with short visit times.

Overall, visits were a mean of 17.4 minutes, which is nearly identical to what ob/gyn physicians reported in a recent survey as their mean time spent with a patient in a postpartum visit.^21^ However, that survey also found nurse-midwives and family medicine physicians reported spending substantially more time with patients (25-28 minutes) than did ob/gyn physicians, suggesting that 17 minutes may be a short time in which to deliver all recommended services associated with a comprehensive postpartum visit. For the most part, differences in services and counseling provided did not significantly differ between those with Medicaid and non-Medicaid insurance types, with the exception of pelvic and breast examinations, which were less common among visits for women with Medicaid in unadjusted (and adjusted, for breast examinations) findings. This finding is similar to previous findings for the content of primary care visits, in which women with Medicaid were less likely to have clinical breast examinations, pelvic examinations, and Papanicolaou tests.^25^ Generally, visits covered by Medicaid had more counseling related to exercise, stress management, and weight reduction, although none of these differences were statistically significant. This lack of statistically significant differences reflects in part the small percentage of visits that included certain recommended services.

Recent research has shown discordance between the care that clinicians consider to be high priority and the care they actual provide.^21^ Similar to previous findings,^21^ we found pelvic examinations were relatively common among the services evaluated, with nearly half of postpartum visits including a pelvic examination. As noted above, this was one of the few services for which we observed differences by insurance status, with pelvic examinations more frequent among women with non-Medicaid insurance (nonsignificant adjusted difference). Evidence of high clinical value for pelvic examinations for asymptomatic women who are not pregnant is lacking; as a result, recent guidelines recommend pelvic examinations for women with particular symptoms or medical histories,^33^ with shared decision making for women without these indications. However, some symptoms that would prompt a pelvic examination, such as dyspareunia and urinary issues, are more common in the postpartum period,^33,34,35,36^ potentially making indicated pelvic examinations more likely.

Despite increased awareness of perinatal depression in recent decades and policy initiatives to promote universal screening,^37,38,39,40,41^ only 8.7% of visits reported depression screening. This finding is consistent with other studies documenting suboptimal rates of postpartum depression screening,^42,43,44^ but in stark contrast to clinician-reported depression screening practices during postpartum visits, because most clinicians report always screening for depression.^21^ Initiatives over time have aimed at improving rates of depression screening, including screening mandates by several states^38^ and work by health systems and practices to ensure more consistent screening protocols.^39^ However, our results indicate an ongoing need to monitor whether this depression screening is conducted and recorded in the clinical record, particularly because there was little change over the study period. Although universal screening for perinatal depression is not sufficient to address perinatal depression, screening is a necessary first step in identifying women in need of treatment.^45,46^ Many hospitals now screen for perinatal depression during the birth hospitalization, but given the natural course of onset and high rates of postpartum depression,^47^ including screening during the postpartum visits with a validated instrument and connecting women to resources is key to ensuring women receive appropriate care.

Contraceptive counseling or provision was included in 43.8% of postpartum visits. Contraceptive counseling and provision is important because of the potential negative health effects associated with a short interpregnancy interval.^48,49,50^ Some contraceptive counseling may occur during prenatal care as ACOG guidelines recommend^6^ or earlier than the comprehensive postpartum visit.^51^ A small minority of women may have received immediate postpartum long-acting, reversible contraception,^52,53,54,55,56,57^ eliminating the need for contraceptive counseling or provision at the postpartum visit. However, this is unlikely to account for the more than half of visits in which contraceptive counseling or provision did not occur.

We found some clinical activities, such as blood pressure monitoring, appear to be near universal, with blood pressure measurements documented at 91.1% of comprehensive postpartum visits. Although blood pressure is likely a vital sign assessed at most clinical visits, measuring blood pressure may alert clinicians to ongoing or acute issues related to hypertensive disorders of pregnancy. This includes the rare possibility of postpartum preeclampsia^58,59^ as well as the more common long-term maternal health risks of blood pressures remaining high in the postpartum period and transitioning into chronic hypertension.^60,61^

### Limitations

Our study had several limitations. First, NAMCS includes only visits to office-based physicians (and in 2014-2016, hospital-employed physicians) and excludes visits to hospital outpatient departments, community health centers, and certified nurse midwives practicing independently. In the last year in which estimates are available (2011), studies estimated approximately 14% of prenatal visits were in hospital outpatient departments,^62,63^ similar to other types of care.^64^ A small percentage of prenatal care is delivered in community health centers.^62,65,66^ Postpartum care is generally provided by the same clinician or clinician group as prenatal care, and so a similar rate of visits provided by non–office-based physicians is expected in this sample. Although a smaller proportion of visits in our sample are Medicaid insured (34.3%) relative to the 43% to 45% of US births covered by Medicaid during this period,^3,22^ women with Medicaid insurance have lower rates of postpartum visit attendance compared with privately insured women,^10,15,16,30,67^ so a lower proportion of Medicaid-insured visits is to be expected in a visit-level sample. Second, NAMCS was shown to have high specificity but low sensitivity for health behavior counseling^68^; our estimates of counseling activities are thus likely underestimates of counseling delivered during postpartum visits.^68^ This research also showed visit times were overestimated in NAMCS vs direct observation, and so visit times we observe may be longer than actual visit times. Third, we are unable to capture all aspects of a comprehensive postpartum visit, such as counseling for women who experienced pregnancy complications and/or follow-up for chronic disease management. In addition, some care may be provided outside of the comprehensive postpartum visits and thus not be captured during this visit (K. H. Geissler, PhD, Jessica Pearlman, PhD, L. Attanasio, PhD; unpublished data; June 2020).^42^ However, service provision during a postpartum visit is essential for improving care during the postpartum period. Fourth, owing to the NAMCS sample size, our study may be underpowered to detect small to medium differences in outcomes between insurance types, particularly for less commonly provided recommended services. However, our results indicate that large differences in care provision for outcomes in recommended services received during office-based physician visits between women insured by Medicaid and other types are unlikely.

## Conclusions

The recent shift toward viewing the postpartum period as a time in which women may need a range of supports to manage their health and that of their new child, rather than limiting postpartum care to a single comprehensive visit, may improve the health care women receive during this period.^6^ In this cross-sectional study, we found most recommended services were provided during less than half of comprehensive postpartum visits and that rates of recommended service provision did not vary by insurance type. Our quantification of recommended service provision during comprehensive postpartum visits will provide a baseline against which to assess potential changes after the ACOG’s 2018 recommendations. Future work should examine the full spectrum of care during the postpartum period to examine ways to improve care provision during this time.

## Notes

Supplement. **eTable 1.** Codes Used in Construction of Contraceptive Counseling Variable **eTable 2.** Full Regression Results for Adjusted Results **eFigure 1.** Unadjusted and Adjusted Estimates for Postpartum Visits With Service Provided **eFigure 2.** Services Provided During Comprehensive Postpartum Visits Over Time (2009-2016)
