# Supplementary material for: Association of Insurance Status With Provision of Recommended Services During Comprehensive Postpartum Visits
Source: JAMA Netw Open. 2020 Nov 10;3(11):e2025095. doi: 10.1001/jamanetworkopen.2020.25095 (PMC7656283; doi:10.1001/jamanetworkopen.2020.25095)
Supplement: Supplement. — eTable 1. Codes Used in Construction of Contraceptive Counseling Variable eTable 2. Full Regression Results for Adjusted Results eFigure 1. Unadjusted and Adjusted Estimates for Postpartum Visits With Service Provided eFigure 2. Services Provided During Comprehensive Postpartum Visits Over Time (2009-2016) [file jamanetwopen-e2025095-s001.pdf]

Supplementary Online Content

Geissler K, Ranchoff BL, Cooper MI, Attanasio LB. Association of insurance status with provision of recommended services during comprehensive postpartum visits. *JAMA Netw Open*. 2020;3(11):e2025095. doi:10.1001/jamanetworkopen.2020.25095

eTable 1. Codes Used in Construction of Contraceptive Counseling Variable
eTable 2. Full Regression Results for Adjusted Results
eFigure 1. Unadjusted and Adjusted Estimates for Postpartum Visits With Service Provided
eFigure 2. Services Provided During Comprehensive Postpartum Visits Over Time (2009-2016)

This supplementary material has been provided by the authors to give readers additional information about their work.

©2020 Geissler K et al. *JAMA Network Open*.

**eTable 1. Codes Used in Construction of Contraceptive Counseling Variable**	
Medications[a]	
a10385	Ethinyl estradiol; iron preparations; norethindrone
a10452	Lactose-norethindrone
c00102	Contraceptives
d00284	Medroxyprogesterone
d00555	Norethindrone
d00557	Levonorgestrel
d01242	Nonoxynol 9 topical
d03238	Ethinyl estradiol-norethindrone
d03241	Ethinyl estradiol-norgestrel
d03242	Ethinyl estradiol-levonorgestrel
d03781	Ethinyl estradiol-norgestimate
d03782	Desogestrel-ethinyl estradiol
d04760	Drospirenone-ethinyl estradiol
d04772	Etonogestrel
d04773	Ethinyl estradiol-etonogestrel
d04779	Ethinyl estradiol-norelgestromin
Reason for visit codes	
3500.0	Family planning NOS
3505.0	Contraceptive medication
3510.0	Diaphragm or IUD insertion, removal or checkup
2525.0	Sterilization or sterilization reversal performed at this visit
4520.0	Norplant insertion/removal
ICD-9 Diagnostic Codes	
V2501	General counseling on prescription of oral contraceptives
V2502	General counseling on initiation of other contraceptive measures
V2503	Encounter for emergency contraceptive counseling and prescription
V2504	Counseling and instruction in natural family planning to avoid pregnancy
V2509	Other general counseling and advice on contraceptive management
V2511	Encounter for insertion of intrauterine contraceptive device
V2512	Encounter for removal of intrauterine contraceptive device
V2513	Encounter for removal and reinsertion of intrauterine contraceptive device
V252	Sterilization
V2540	Contraceptive surveillance, unspecified
V2541	Surveillance of contraceptive pill
V2542	Surveillance of intrauterine contraceptive device
V2543	Surveillance of implantable subdermal contraceptive
V2549	Surveillance of other contraceptive method

©2020 Geissler K et al. *JAMA Network Open*.

V255	Insertion of implantable subdermal contraceptive
V258	Other specified contraceptive management
V259	Unspecified contraceptive management
V260	Tuboplasty or vasoplasty after previous sterilization
V2641	Procreative counseling and advice using natural family planning
V2651	Tubal ligation status
V2652	Vasectomy status
ICD-10 Diagnostic Codes	
Z30.0	Encounter for general counseling and advice on contraception
Z30.01	Encounter for initial prescription of contraceptives (includes pills, emergency contraception, injectable contraception, IUD, vaginal ring, transdermal patch, implantable subdermal contraceptives, other)
Z30.02	Counseling and instruction in natural family planning to avoid pregnancy
Z30.09	Encounter for other general counseling and advice on contraception
Z30.2	Encounter for sterilization
Z30.4	Encounter for surveillance of contraceptives (includes pills, emergency contraception, injectable contraception, IUD, vaginal ring ,transdermal patch, implantable subdermal contraceptives, other)
Z30.8	Encounter for other contraceptive management
Z30.9	Encounter for contraceptive management, unspecified
ICD-9 Procedure Codes	
697	Insertion of intrauterine contraceptive device

Note: [a] The question for medications changed from "were medications or immunizations ordered or provided at this visit?" (2009-2011) to "were any prescription or non-prescription drugs ordered or provided (by any route of administration) at this visit?" This list of medications for contraceptives is not fully inclusive – it is limited to contraceptives that were provided during at least one comprehensive postpartum visit in the 2009 to 2016 period.

©2020 Geissler K et al. *JAMA Network Open*.

eTable 2. Full Regression Results for Adjusted Results

Outcome variable	Blood pressure taken	Depression screening	Pelvic exam	Pap test	Breast exam	Contraceptive counseling or provision	Counseling - weight reduction	Counseling - exercise	Counseling - stress management	Counseling - diet/nutrition	Medication ordered or provided	Refer to other physician
Medicaid insurance	0.0303	-0.0130	-0.0994	0.00863	-0.108**	0.00800	0.0221	0.0519	0.0569	-0.00185	-0.0367	-0.0160
	(0.0357)	(0.0342)	(0.0628)	(0.0444)	(0.0462)	(0.0658)	(0.0225)	(0.0593)	(0.0485)	(0.0601)	(0.0578)	(0.0174)
Patient age in years	0.00269	0.00199	-0.00288	-0.000990	-0.00321	-0.0118***	3.11e-06	0.00519*	0.00281*	0.00235	-0.00854*	-0.00129
	(0.00262)	(0.00315)	(0.00426)	(0.00407)	(0.00368)	(0.00421)	(0.00133)	(0.00278)	(0.00160)	(0.00267)	(0.00443)	(0.00112)
Race/Ethnicity (reference group = Non-Hispanic White)												
Non-Hispanic Black	0.0297	0.0718	-0.0118	-0.0672	0.0496	0.0234	-0.00696	-0.0201	-0.00786	0.0312	-0.107	0.0342
	(0.0340)	(0.0558)	(0.0685)	(0.0463)	(0.0672)	(0.0703)	(0.0377)	(0.0483)	(0.0366)	(0.0583)	(0.0673)	(0.0278)
Hispanic	0.0142	0.0306	-0.0255	-0.0731	-0.0159	0.0168	-0.0232	-0.0122	-0.0450	0.0104	0.0603	-0.000931
	(0.0393)	(0.0358)	(0.0725)	(0.0507)	(0.0542)	(0.0708)	(0.0293)	(0.0442)	(0.0308)	(0.0417)	(0.0608)	(0.0120)
Non-Hispanic Other	0.0521	0.0751	-0.0696	-0.0833*	-0.0660	-0.0537	-0.0354*	-0.0324	0.0586	0.0659	-0.00340	0.0783
	(0.0333)	(0.0882)	(0.111)	(0.0477)	(0.0709)	(0.100)	(0.0200)	(0.0427)	(0.0690)	(0.0712)	(0.0857)	(0.0599)
Patient Comorbidities												
Asthma	0.0520	0.00586	-0.176*	-0.105*	-0.0121	-0.210***	-0.0626*	0.0182	0.0748	0.00176	0.0961	-0.0521*
	(0.0361)	(0.0802)	(0.105)	(0.0577)	(0.106)	(0.0795)	(0.0343)	(0.0847)	(0.0797)	(0.0934)	(0.0918)	(0.0303)
Diabetes	0.0177	-0.0675**	-0.0401	0.156	-0.207***	-0.165	0.0597	-0.1000***	-0.0176	-0.0737	-0.327**	-0.0319
	(0.0721)	(0.0308)	(0.110)	(0.108)	(0.0634)	(0.141)	(0.0797)	(0.0344)	(0.0269)	(0.0672)	(0.127)	(0.0237)
Depression	0.0318	0.300***	-0.115	0.0151	-0.0678	-0.0934	-0.0152	0.0432	0.113*	-0.00192	0.0867	0.0806*
	(0.0359)	(0.0972)	(0.0796)	(0.0576)	(0.0705)	(0.0795)	(0.0248)	(0.0575)	(0.0636)	(0.0529)	(0.0817)	(0.0442)
Hypertension	0.00754	-0.0599	-0.162*	0.0112	0.140	-0.158*	0.114	0.0978	0.00836	0.193*	0.286***	0.0475
	(0.0353)	(0.0608)	(0.0966)	(0.0693)	(0.100)	(0.0930)	(0.0713)	(0.0732)	(0.0494)	(0.0989)	(0.0821)	(0.0647)
Obesity	0.00640	-0.0117	0.236**	-0.0856	-0.0728	0.168	-0.0349	-0.0509	0.0912	-0.00885	0.0939	0.00683
	(0.0601)	(0.0420)	(0.102)	(0.0544)	(0.0838)	(0.111)	(0.0232)	(0.0520)	(0.0782)	(0.0775)	(0.113)	(0.0220)
Physician is OB/GYN (versus family medicine)	-0.105***	0.0102	0.213***	-0.0563	0.0516	0.0743	-0.0652	0.0268	0.0395	-0.00339	0.114	0.0292
	(0.0352)	(0.0662)	(0.0704)	(0.0752)	(0.0719)	(0.105)	(0.0482)	(0.0523)	(0.0426)	(0.0766)	(0.133)	(0.0179)

©2020 Geissler K et al. *JAMA Network Open*.

Physician office located in metropolitan statistical area	0.0822	0.0129	0.0863	-0.103	-0.0187	0.0705	0.0176	-0.104*	-0.0366	-0.0959	0.0522	0.00714
	(0.0523)	(0.0491)	(0.0683)	(0.0701)	(0.0591)	(0.0983)	(0.0271)	(0.0615)	(0.0377)	(0.0742)	(0.0994)	(0.0110)
Physician is full or part owner of practice	0.0197	-0.0662*	0.0317	0.0655	-0.0208	0.0533	-0.000275	0.0646*	0.0265	0.0241	-0.0203	-0.0118
	(0.0336)	(0.0353)	(0.0606)	(0.0445)	(0.0507)	(0.0545)	(0.0231)	(0.0342)	(0.0261)	(0.0373)	(0.0564)	(0.0160)
Office setting is a private solo or group practice	0.00492	-0.0352	-0.128	0.0471	0.0849	-0.0366	0.0300*	-0.112**	-0.0997	-0.131**	-0.0860	0.0441**
	(0.0584)	(0.0820)	(0.0955)	(0.0491)	(0.0641)	(0.0641)	(0.0161)	(0.0559)	(0.0633)	(0.0567)	(0.0979)	(0.0191)
Physician is in a Solo Practice	0.00558	-0.0135	-0.0608	-0.0414	-0.0497	-0.182**	-0.00974	-0.00565	0.0169	0.000189	-0.0744	-0.0262*
	(0.0399)	(0.0305)	(0.0716)	(0.0483)	(0.0441)	(0.0713)	(0.0169)	(0.0344)	(0.0276)	(0.0366)	(0.0604)	(0.0138)
Practice uses any electronic medical records	-0.0626*	0.0238	0.0905	0.00796	0.130***	-0.0380	-0.00268	0.00127	-0.0289	-0.00697	0.125**	0.0102
	(0.0340)	(0.0275)	(0.0644)	(0.0487)	(0.0429)	(0.0674)	(0.0222)	(0.0355)	(0.0328)	(0.0371)	(0.0598)	(0.0133)
Year	0.0179**	-0.00149	-0.0251*	-0.0308***	-0.0316***	-0.0213*	-0.00691*	-0.0108	0.000581	0.00144	0.0241*	-0.00166
	(0.00718)	(0.00813)	(0.0139)	(0.00966)	(0.00995)	(0.0117)	(0.00368)	(0.00656)	(0.00488)	(0.00759)	(0.0130)	(0.00331)
Number of visits (weighted)	20,071,093	20,071,093	20,071,093	20,071,093	20,071,093	20,071,093	20,071,093	20,071,093	20,071,093	20,071,093	20,013,231	19,397,174
Number of visits (unweighted)	645	645	645	645	645	645	645	645	645	645	642	627

Note:

Standard errors in parentheses

*** p<0.01, ** p<0.05, * p<0.10

©2020 Geissler K et al. *JAMA Network Open*.

eFigure 1. Unadjusted and Adjusted Estimates for Postpartum Visits With Service Provided

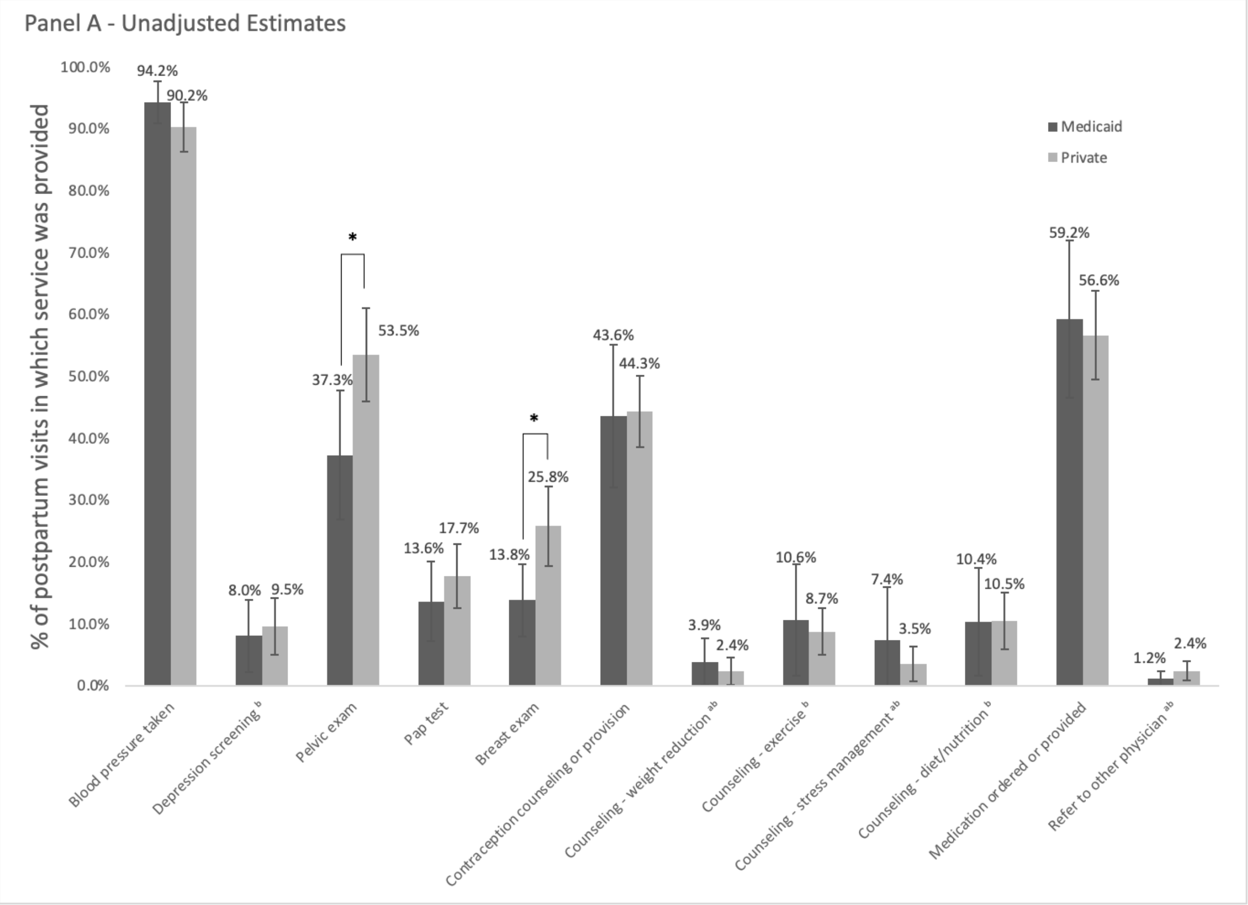

Note: * indicates difference between Medicaid and private insurance estimates is statistically significant with p<0.05.

95% confidence intervals are shown with vertical bars. Sample size is 19,186,401 visits (n=614 unweighted). Regression adjusted estimates of predicted probabilities are reported; controls are included for whether the visit was paid by Medicaid, year of visit, patient age, patient race/ethnicity, patient comorbidities (asthma, diabetes, depression, hypertension, obesity), physician specialty (OB/GYN versus family medicine), office location in a metropolitan statistical area, physician is full or part owner of practice, private solo or group practice, and solo practice. Standard errors correct for the complex survey design. NAMCS notes that NCHS does not consider estimates relying on fewer than 30 observations and/or with standard errors greater than 30% of estimates to be reliable. The estimates for private insurance adjusted estimates noted with ‘a’ have standard errors (as shown as part of 95% CI) that exceed this threshold, those estimates for Medicaid adjusted estimates with ‘b’ have standard errors that exceed this threshold. These estimates are reported for completeness. Number of weighted visits for referral to other physician is N=18,546,831 (n=599); for medication ordered or provided number of weighted visits is 19,128,538 (n=611).

©2020 Geissler K et al. *JAMA Network Open*.

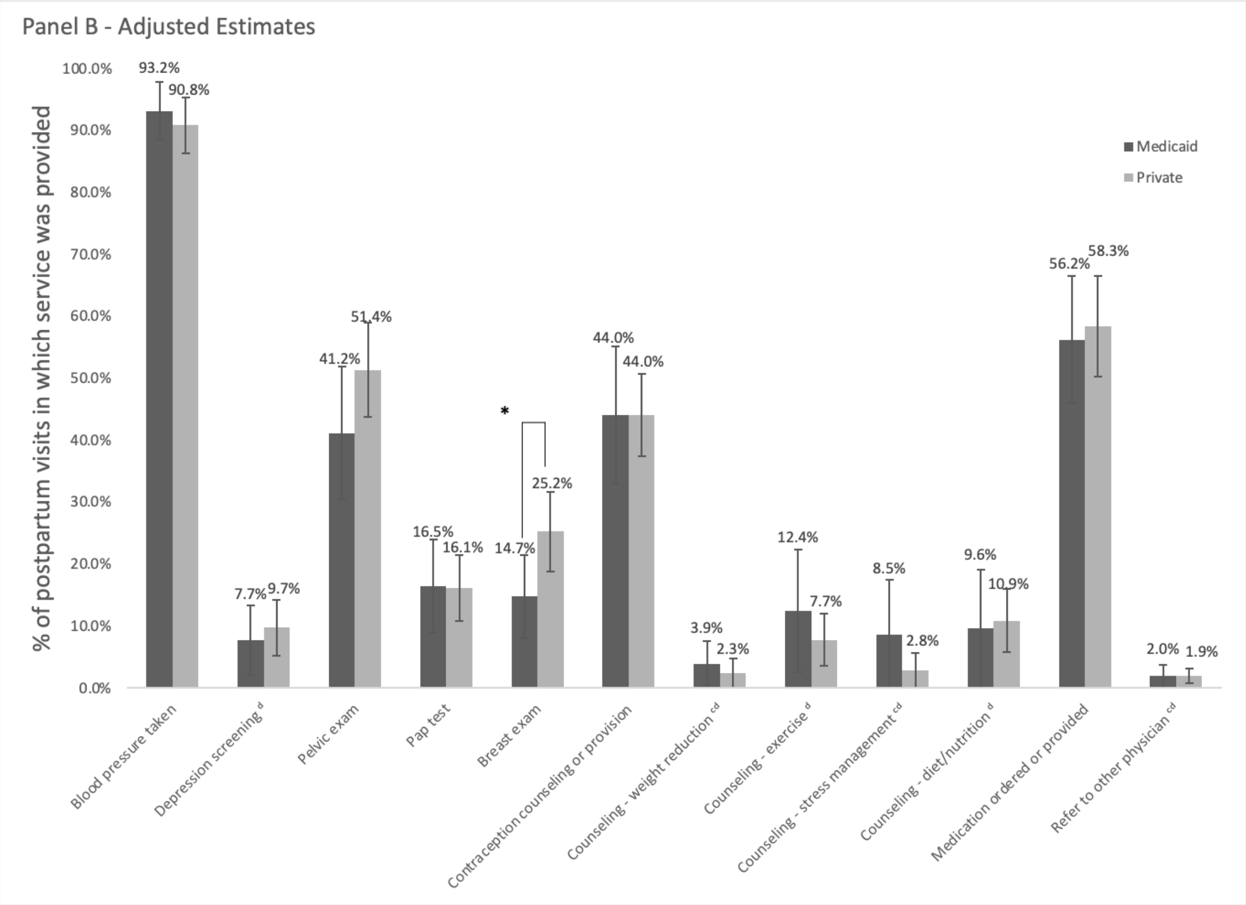

Note: * indicates difference between Medicaid and private insurance estimates is statistically significant with $p<0.05$.

95% confidence intervals are shown with vertical bars. Sample size is 19,186,401 visits (n=614 unweighted). Regression adjusted estimates of predicted probabilities are reported; controls are included for whether the visit was paid by Medicaid, year of visit, patient age, patient race/ethnicity, patient comorbidities (asthma, diabetes, depression, hypertension, obesity), physician specialty (OB/GYN versus family medicine), office location in a metropolitan statistical area, physician is full or part owner of practice, private solo or group practice, and solo practice. Standard errors correct for the complex survey design. NAMCS notes that NCHS does not consider estimates relying on fewer than 30 observations and/or with standard errors greater than 30% of estimates to be reliable. The estimates for private insurance adjusted estimates noted with 'c' have standard errors (as shown as part of 95% CI) that exceed this threshold, those estimates for Medicaid adjusted estimates with 'd' have standard errors that exceed this threshold. These estimates are reported for completeness. Number of weighted visits for referral to other physician is N=18,546,831 (n=599); for medication ordered or provided number of weighted visits is 19,128,538 (n=611).

©2020 Geissler K et al. *JAMA Network Open*.

eFigure 2. Services Provided During Comprehensive Postpartum Visits Over Time (2009-2016)

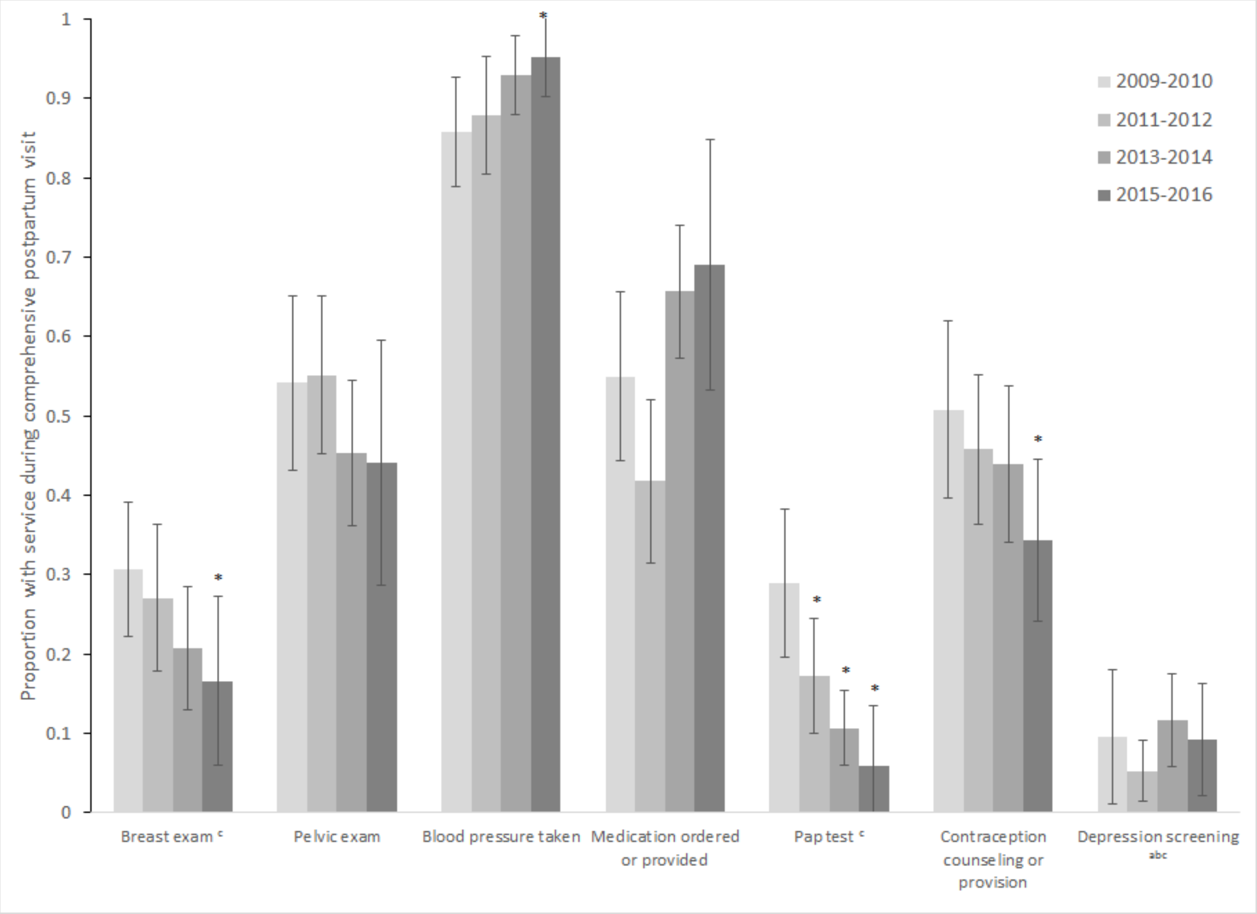

Note: Estimates are predicted probabilities from regression analysis adjusting for Medicaid insurance only. An * indicates estimate is significantly different from 2009-2010 estimate at the 5% level. Standard errors correct for the complex survey design. NAMCS notes that NCHS does not consider estimates relying on fewer than 30 observations and/or with standard errors greater than 30% of estimates to be reliable. The estimates for the 2009-2010 adjusted estimates noted with ‘a’ have standard errors (as shown as part of 95% CI) that exceed this threshold, those estimates for 2011-2012 adjusted estimates noted with ‘b’ have standard errors that exceed this threshold, those estimates for 2015-2016 adjusted estimates with ‘c’ have standard errors that exceed this threshold. These estimates are reported for completeness.

©2020 Geissler K et al. *JAMA Network Open*.
